# Targeting STAT3 in Cancer with Nucleotide Therapeutics

**DOI:** 10.3390/cancers11111681

**Published:** 2019-10-29

**Authors:** Yue-Ting K. Lau, Malini Ramaiyer, Daniel E. Johnson, Jennifer R. Grandis

**Affiliations:** Department Otolaryngology—Head and Neck Surgery, University of California at San Francisco, 1450 3rd Street, Room HD268, Box 3111, San Francisco, CA 94143, USA; Kara.Lau@ucsf.edu (Y.-T.K.L.); malinisramaiyer@gmail.com (M.R.); daniel.johnson@ucsf.edu (D.E.J.)

**Keywords:** hedging, transaction costs, dynamic programming, risk management, post-decision state variable

## Abstract

Signal transducer and activator of transcription 3 (STAT3) plays a critical role in promoting the proliferation and survival of tumor cells. As a ubiquitously-expressed transcription factor, STAT3 has commonly been considered an “undruggable” target for therapy; thus, much research has focused on targeting upstream pathways to reduce the expression or phosphorylation/activation of STAT3 in tumor cells. Recently, however, novel approaches have been developed to directly inhibit STAT3 in human cancers, in the hope of reducing the survival and proliferation of tumor cells. Several of these agents are nucleic acid-based, including the antisense molecule AZD9150, CpG-coupled STAT3 siRNA, G-quartet oligodeoxynucleotides (GQ-ODNs), and STAT3 decoys. While the AZD9150 and CpG-STAT3 siRNA interfere with STAT3 expression, STAT3 decoys and GQ-ODNs target constitutively activated STAT3 and modulate its ability to bind to target genes. Both STAT3 decoy and AZD9150 have advanced to clinical testing in humans. Here we will review the current understanding of the structures, mechanisms, and potential clinical utilities of the nucleic acid-based STAT3 inhibitors.

## 1. Introduction

Signal transducer and activator of transcription 3 (STAT3) is a transcription factor that is overexpressed and/or hyperactivated in multiple human cancers, where it enhances tumor cell survival and invasion through transcription of anti-apoptotic and pro-proliferative genes [1]. STAT3 has also been shown to directly interact with mitochondrial DNA to contribute to Ras-dependent malignant transformation and cancer progression by amplifying electron transport chain function [2,3,4,5,6]. STAT3 overexpression has been significantly associated with poor overall survival rates in patients with solid tumors [7]. Aberrant phosphorylation of STAT3 on Tyrosine 705 or Serine 727 by upstream kinases results in hyperactivation of the STAT3 protein [8,9]. In addition, genome silencing of phosphatases that play a role in dephosphorylation/inactivation of STAT3, such as those encoded by *PTPR* genes [10], can also result in constitutive activation of STAT3 in cancer [11].

In addition to contributing to the proliferation and survival of tumor cells, STAT3 hyperactivation plays an important role in the resistance of tumors to conventional chemotherapy drugs, as well as molecular targeting agents [12]. Moreover, STAT3 promotes immunosuppression in the tumor microenvironment [13]. STAT3 activation in tumor cells leads to increased production of immunosuppressive cytokines, including interleukin-6 (IL-6), IL-10, vascular endothelial growth factor (VEGF), and transforming growth factor-b1 (TGF-β1) [14,15,16,17]. Cytokines and growth factors produced by tumor cells also commonly lead to STAT3 activation in tumor-infiltrating immune cells. The activation of STAT3 in infiltrating immune cells, in general, inhibits anti-tumor immunity. Specifically, STAT3 exhibits cell-autonomous inhibitory activities against cytotoxic T cells (CTLs), natural killer (NK) cells, and dendritic cells (DCs), while increasing the levels of immunosuppressive T regulatory (Treg) and myeloid-derived suppressor cells (MDSCs) [18].

**Figure 1:**
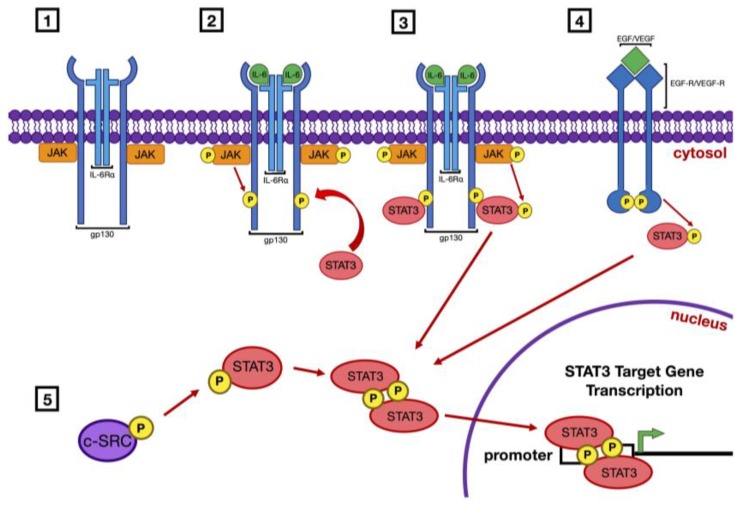
Pathways of signal transducer and activator of transcription 3 (STAT3) activation. Activation of STAT3 occurs via the initial phosphorylation of tyrosine 705 on the STAT3 molecule. This can occur in several ways: (1) Ligand binding activates Janus kinases (JAKs) that are associated with a receptor that lacks intrinsic tyrosine kinase activity, such as the interleukin-6 (IL-6) receptor/gp130 complex; (2) Activated JAKs phosphorylate the cytoplasmic region of the receptor molecule which then serves as a recruitment site for STAT3; (3) STAT3 is then phosphorylated by JAK. (4) Receptor tyrosine kinases (RTK) such as epidermal growth factor receptor (EGFR) or vascular endothelial growth factor receptor (VEGFR) have intrinsic kinase capabilities which directly phosphorylate STAT3 following ligand binding. (5) Receptor independent tyrosine kinases such as c–SRC can phosphorylate JAK without receptor activation. Once Y705 of STAT3 is phosphorylated the SH2 domain of each STAT3 molecule binds the phospho-tyrosine of another, resulting in dimerization of the two proteins. These homodimers are then able to translocate to the nucleus and bind the promoter regions of target genes and induce their transcription. Many of these target genes encode proteins that drive cellular proliferation and survival.

Activation of STAT3 is known to occur via several general pathways (Figure 1). Ligand binding initiates engagement of receptor tyrosine kinases, such as the receptors for epidermal growth factor (EGF) or vascular endothelial growth factor (VEGF), or receptors that lack intrinsic tyrosine kinase activity, such as the IL-6 receptor/gp130 complex, and/or nonreceptor tyrosine kinases such as c-Src [19].

In the case of receptors lacking kinase activity, the binding of ligand leads to activation of receptor-associated Janus kinases (JAK). Activated JAKs phosphorylate the cytoplasmic region of the receptor molecule, which then serves as a docking site for STAT3. Recruitment of STAT3 then results in the direct phosphorylation of Tyrosine 705 by JAK. Phosphorylation of Serine 727 has also been shown to occur secondarily as a mechanism for maximal activation [20]. Receptor tyrosine kinases, including EGFR and VEGFR, harbor intrinsic kinase activity. Following ligand binding, the cytoplasmic regions of receptor tyrosine kinases are subjected to autophosphorylation, and these sites of phosphorylation then serve to recruit STAT3 which is subsequently phosphorylated/activated by the activated receptor.

Phosphorylation of Tyrosine 705 leads to homodimerization of STAT3 proteins, but STAT3 can also participate in alternative heterodimerization with STAT1α [21,22]. Canonical homodimerization occurs via SH2 recognition of the phospho-tyrosine residue in another STAT3 molecule and subsequent binding. STAT3 homodimers translocate to the nucleus where they bind to the promoter regions of STAT3 target genes, inducing the transcription of a broad number of genes whose products drive cellular proliferation and survival. STAT3 has also been demonstrated in an important role as a transcription modulator for mitochondrial respiration and oxidative metabolism [23,24,25].

### 1.1. Peptide and Small Molecule Inhibitors of STAT3

The critical role that overexpression and/or hyperactivation of STAT3 plays in the development of multiple cancers has spawned considerable effort to develop inhibitory molecules with potential for clinical application. Early efforts were focused on the development of peptide-based inhibitors. Additional pursuits have led to the identification of several small molecule inhibitors of STAT3. Furthermore, natural derivatives and natural models as lead scaffolds for molecular design have recently gained ground and demonstrated efficacy in STAT3 inhibition. In general, both peptidic inhibitors and small molecule inhibitors of STAT3 suffer from issues of low potency, poor cell penetrance, or undesirable nonspecific activities. However, a few recently discovered small molecule inhibitors are showing considerable promise and have reached the stage of advancing to clinical testing [26]. Hence, before describing nucleic acid-based inhibitors of STAT3 we will briefly review progress made in developing peptide and small molecule inhibitors.

### 1.2. Peptide Inhibitors

Critical to the function of STAT3 is the recognition of phosphotyrosine residues on activated cell surface receptors (for the purpose of binding to the receptor), or phosphotyrosine 705 on another STAT3 molecule (for the purpose of homodimerization). Recognition of these phosphotyrosine residues occurs via the STAT3 SH2 domain, which recognizes the consensus sequence PY*LKTK (where Y* represents phosphotyrosine). Early studies by Turkson et al. demonstrated that phosphorylated PY*LKTK peptide inhibited the DNA binding activity of STAT3 in nuclear extracts, but had no activity against STAT5 [27]. However, exceptionally high concentrations of the phosphorylated peptide were required to inhibit STAT3 activity in cells. In an effort to generate a more stable version of the PY*LKTK peptide, a peptidomimetic version named ISS-610 was developed and shown to have improved capacity for inhibiting STAT3 DNA binding activity in NIH3T3 cells (IC_50_ = 42 µM) [28]. ISS-610 also demonstrated growth inhibitory activity against several different cancer cell lines characterized by hyperactivation of STAT3. Mandal et al. made additional modifications in an effort to prevent dephophorylation of the peptide, generating the agent PM-73G [29]. This novel drug disrupted STAT3 DNA binding activity in the nanomolar range and also slowed the growth of MDA-MB-468 tumor xenografts.

Despite advances made in generating peptide and peptidomimetic inhibitors of STAT3, further development is hindered by multiple factors. The potencies, stabilities, and cellular uptake of peptides and peptidomimetic compounds will need to be improved and specificities will need to be closely evaluated. Moreover, there is concern that peptides and peptidomimetics may stimulate an immune response, which could limit their effectiveness.

### 1.3. Small Molecule Inhibitors

Given the obstacles associated with developing clinically relevant peptide inhibitors, greater emphasis has been placed on identifying small molecule inhibitors of STAT3 [30,31]. A number of small-molecule STAT3 inhibitors have been discovered using either experimental screening strategies or virtual screening approaches. Among the best characterized small molecule inhibitors are STATTIC, OPB-31121, OPB-51602, OPB-111077, and C188-9. In addition, an alternative pathway in mitochondrial STAT3 inhibition has been described in the small molecule MDC-1112.

STATTIC was identified in a screen of 17,000 compounds that used a fluorescence polarization assay to detect compounds capable of dissociating phosphopeptide from the SH2 domain of STAT3 [29,32]. STATTIC inhibits STAT3 DNA binding activity and slows the growth of xenograft tumors representing breast cancer and head and neck squamous cell carcinoma [31]. Treatment with STATTIC has been shown to enhance the activities of chemotherapy and radiation against cancer cells in vitro [33].

OPB-31121, OPB-51602, and OPB-111077 are orally bioavailable inhibitors developed by Otsuka Pharmaceutical Company. Molecular and computational modeling studies indicate high affinity binding (Kd = 10 nM) of these compounds to the SH2 domain of STAT3 [34]. Preclinical studies with OPB-31121 have shown that the compound inhibits STAT3 DNA binding and enhances the activities of chemotherapy drugs in leukemia and gastric cancer models [34,35,36]. OPB-31121 also slows the growth of primary leukemia and SNU484 gastric cancer xenograft tumors in mice [35,36]. A Phase I trial of OPB-31121 revealed considerable dose-limiting toxicities at doses below those needed for STAT3 inhibition. Furthermore, limited anti-tumor activity was observed [37]. Similar to OPB-31121, OPB-51602 demonstrates growth inhibition against xenograft tumors in preclinical models [38]. Phase I testing of OPB-51602 demonstrated reduction of phosphorylated STAT3 in monocytic cells and regression of tumors in two patients with non-small cell lung cancer [39]. One of these patients exhibited complete regression of target lesions and a progression-free survival interval of 6.9 months, while the other responder showed a 41 percent decline in tumor burden. However, treatment with OPB-51602 for multiple cycles resulted in substantial toxicities in multiple patients, including diarrhea, nausea, lactic acidosis, and peripheral neuropathy, leading to necessary discontinuation of treatment [38,39]. Phase I evaluation of OPB-111077 has demonstrated greater tolerability, with only mild or moderate side effects. To date, only modest anti-tumor responses have been observed with the OPB-111077, and further clinical investigation is necessary.

The small molecule C188 was discovered via virtual screening of a large compound library (920,000 compounds) to identify small molecules that may bind to the STAT3 SH2 domain [40]. Evaluation of C188 in cell-free assays demonstrated its ability to abrogate binding of phosphopeptide to the SH2 domain. Chemical optimization of C188 yielded the compound C188-9, also called TTI-101, with the capacity to inhibit phosphopeptide/SH2 interactions in the low nanomolar range [41]. C188-9 is an orally bioavailable STAT3 inhibitor that exhibits in vitro and in vivo activity against preclinical models of non-small cell lung cancer [42], head and neck squamous cell carcinoma [41], liver cancer [43], and breast cancer [44]. Phase I evaluation of C188-9 in patients with solid tumors is currently underway.

MDC-1112, or V-P, is a valporic acid derivative synthesized by Medicon Pharmaceuticals Inc. which blocks the translocation of STAT3 into the mitochondria and promotes the production of reactive oxygen species, inducing cellular apoptosis [45]. In combination with cimetidine, MDC-1112 inhibited the growth of pancreatic cancer xenografts in mice by 60–70% [45] and glioblastoma multiforme xenografts by 78.2% [46].

Napabucasin (2-acetylfuro-1,4-naphthoquinone or BBI-608) is a small molecule in clinical development that has been reported to abrogate STAT3 signaling. A small Phase I trial in combination with paclitaxel for advanced/recurrent gastric cancer reported that the combination was well tolerated [47]. A Phase III trial investigating napabucassin in combination with nab-paclitaxel and gemcitabine for metastatic pancreatic cancer is ongoing [48].

### 1.4. Future Directions for Small Molecule STAT3 Inhibitors

Although current small molecule inhibitors of STAT3, particularly C188-9, hold potential promise, the success rate for generating clinically viable small molecule inhibitors is low. A number of factors contribute to this low rate of success, including poor pharmacokinetic properties, insufficient potency, and unacceptable levels of nonspecific activity leading to toxic side effects. In addition, acquired resistance to small molecule inhibitors frequently occurs via mutation of the binding site on the target protein. A new approach towards drug development using molecules called proteolysis-targeting chimeras (PROTACs) may help to overcome some of the issues related to nonspecific activity and acquired resistance [49,50]. In the PROTACs approach, a fusion molecule is generated in which a linker sequence is used to covalently connect a small molecule inhibitor to a molecule (eg., thalidomide) that can attract an intracellular E3 ubiquitin ligase. By bringing the E3 ligase in close proximity to the target protein (eg., STAT3), the inhibitor fusion molecule serves to facilitate ubiquitination and subsequent proteasomal degradation of the target protein [49,50]. This process is both rapid and catalytic, meaning the fusion inhibitor molecule is released after destruction of the target protein and can subsequently interact and facilitate destruction of additional target molecules. Because of this catalytic nature, low doses of the inhibitor fusion molecule can be used to achieve efficient removal of the target protein, minimizing the impact of nonspecific activities that would be seen when higher doses are required for target inhibition by a conventional small molecule inhibitor. Moreover, because elimination of the target protein occurs rapidly, the risk of developing acquired resistance via target protein mutation is minimized. The application of PROTACs to inhibition of STAT3 signaling is an exciting opportunity, although, to date, no STAT3 PROTACs inhibitors have been reported.

### 1.5. Natural Inhibitors

Of the currently available anti-cancer agents used in current practice, it was found that over 40% were based in or derived from natural products [51]. Because of the increasing development of resistance to chemotherapeutic drugs, novel strategies to generate treatments can be sought in bioprospecting and combinatorial biosynthesis from nature-based derivatives. Recent advances in plant-based derivatives that have shown efficacy in STAT3 inhibition include Erasin, bruceanitol, and Curcumin.

Erasin is a chromone-based STAT5 inhibitor that has hydrophobic substituents at the 6-position, resulting in STAT3 specific inhibition. Derived from the natural product classes of flavones and isoflavones, it was determined to decrease STAT3 Y705 phosphorylation and increase apoptosis in MDA-MB-231 breast cancer cells, as well as Erlotinib resistant non-small cell lung cancer (NSCLC) cells [52].

From Brucinea javanica, a chinese plant used to treat cancer, bruceanitol (BOL) was isolated and discovered to have potent anti-leukemic activity. BOL demonstrated the ability to suppress cell growth in a wide range of genetically varied colorectal cancer cell lines, all of which were equally sensitive to the compound [53]. Reductions in phosphorylated STAT3 and downstream target expression of Mcl-1, c-Myc, and Survivin was observed at BOL concentrations of 30 nM. In vivo experiments on mice with CRC xenografts reflected similar results with doses of 2 mg/kg and 4 mg/kg reducing p-STAT3 32% and 80%, and tumor volume 35% and 58% respectively [53].

Diferuloylmethane is a polyphenol derived from the plant Curcuma longa, known as Curcumin, and has been shown to downregulate the expression of downstream STAT3 expression targets such as BCL-2 and Bcl-xL. Curcumin was shown to downregulate the production of Survivin mRNA concordant with the reduction of p-STAT3 in pancreatic cancer cell lines [54].

Collectively these naturally-derived small molecules offer considerable potential in the inhibition of STAT3 hyperactivation in pre-clinical studies. Natural products serve as promising starting points for development of inhibitors, but represent a platform that is currently difficult to exploit because of the lack of knowledge surrounding lead structures for rational design.

### 1.6. Nucleic Acid-Based Agents to Inhibit Expression of STAT3

#### 1.6.1. AZD9150

Inhibition of STAT3-mediated gene expression can be achieved by directly inhibiting STAT3 expression using antisense oligonucleotides that promote the destruction or inhibit the translation of STAT3 mRNA. These oligonucleotides are short, 12–25 nucleotide strands with sequences such as 5′-GCTCCAGCATCTGCTGCTTC-3′, designed to pair with complementary STAT3 mRNA sequence(s) [55,56]. Binding results in cleavage of the target via RNAse H, alteration of post-transcriptional RNA splicing, or arrest of translation, leading to downmodulation of STAT3 expression [55]. Several generations of antisense oligonucleotides have been developed and chemically modified to optimize stability and allow systemic administration [57].

Early versions of the STAT3 antisense molecule contained 2′-O-methyl or 2′-O-methoxyethyl moieties to prevent free-end degradation and were synthesized with phosphorothioate chemistry to provide further stability [58]. Expression of STAT3 and STAT3 response genes was reduced when prostate (DU145) [59,60], breast (SCK), and melanoma (B16) [61] cell lines were treated with STAT3 antisense oligonucleotides. Furthermore, in vivo experiments showed inhibition of STAT3-mediated tumorigenesis, angiogenesis, and tumor growth in xenograft mouse models of prostate and hepatocellular carcinoma [59,62]. Sensitivity to STAT3 antisense treatment was also demonstrated in androgen-resistant models of prostate cancer and in lung metastases arising from hepatocellular primary tumors [59,62].

The second-generation antisense oligonucleotide AZD9150 (ISIS 481464) is the only STAT3 antisense molecule to enter clinical trials. The ASO structure of AZD9150 was optimized by replacing the 2′-O-methoxyethyl groups with constrained ethyl modifications to increase stability and potency [63]. Preclinical studies with AZD9150 in lymphoma (KARPAS299 and SUP-M2) [63] and neuroblastoma (IMR 32) [64] cell lines with aberrantly activated STAT3 showed a decrease in the expression of STAT3 protein and downstream signaling targets. AZD9150 also demonstrated selective uptake and promoted impaired growth in hematopoietic myelodysplastic and leukemic stem cells [65]. Systemic administration of AZD9150 to immunodeficient mice harboring lymphoma, neuroblastoma, or non-small-cell lung cancer xenografts resulted in decreased STAT3 expression in tumor cells and reduction of tumor initiating potential following serial implantation of the AZD9150-treated tumors [63,64]. While established tumor growth in neuroblastoma xenografts was not inhibited, systemic administration of AZD9150 led to reversal of STAT3-mediated resistance to cisplatin, as indicated by a twofold decrease in the IC_50_ for cisplatin [64].

As a class, antisense oligonucleotides have demonstrated nonspecific immune system activation due to the presence of unmethylated CpG motifs that are recognized as pathogenic [66]. Toxicological effects of AZD9150 have been studied in cynomolgus monkeys and in mice, with key findings being transient prolongation of intrinsic pathway clotting times, elevation of serum transaminase levels, and accumulation in renal and liver tissue [67]. However, these effects occur in doses far greater than those used in clinical trials, with no signs of end organ damage in kidneys or liver until doses of 40 mg/kg or 70 mg/kg, respectively, are reached.

A sufficient therapeutic margin provided the foundation to carry out a phase I clinical trial (NCT01563302) in 30 patients with hematologic and solid malignancies refractory to at least one prior systemic therapy [68]. All responses following administration of AZD9150 at dose levels of 2 mg/kg and 3 mg/kg occurred in diffuse large B cell lymphoma (DLBCL) patients. Two DLBCL patients achieved complete response, two achieved a partial response, and one maintained stable disease for a response rate of 13% [69]. There was no significant difference in progression-free and overall survival between the dose levels. Notable adverse events included transaminitis, fatigue, thrombocytopenia, nausea, and anemia with thrombocytopenia being more closely related to high-grade events. Based on the observed toxicities, the recommended phase 2 trial dose was 3 mg/kg. Peripheral blood analyses in this trial suggested an impact of AZD9150 in selected immune cell populations [69].

The heterogeneity in the response of lymphoma patients, five of whom had DLBCL, to AZD9150 highlights the importance of exploring what factors predict the efficacy of treatment. STAT3 inhibition may increase the immunogenicity of malignant cells through cell-autonomous processes and prevent tumor microenvironment immunosuppression [70]. Immunogenic biomarkers could thus offer clues to treatment progress and success. This could also lay the foundation for examining how combination with immunotherapeutic agents may further increase therapeutic benefits.

#### 1.6.2. CpG-coupled STAT3 siRNA

While STAT3 is known to be overexpressed in tumor cells, it is also dysregulated in tumor-associated myeloid cells, such as dendritic cells and macrophages [71]. This results in a loss of MHCII expression and the accumulation of inactive antigen presenting cells. In effect, there is reduced Th-1-mediated CD8+ cytotoxicity towards tumor cells [13], and an increased presence of myeloid-derived suppressor cells (MDSCs) and regulatory T-cells (Tregs) generating an immunosuppressive tumor microenvironment [72].

Selective targeting of STAT3 in tumor-associated myeloid-derived cells is possible with siRNA conjugation to a CpG TLR-9 ligand [73]. TLR9 is a cell-surface transmembrane receptor that is upregulated under conditions of cellular or environmental stress and is known to be expressed on myeloid-derived cells in the tumor microenvironment [74]. It is also upregulated in acute myeloid leukemia, multiple myeloma, and B-cell lymphoma, and activation has been shown to increase antigenicity of primary malignant B-cells and induce apoptosis [75]. When activated by CpG binding, TLR9 facilitates immunostimulatory signaling and the release of proinflammatory cytokines as well as the presentation of tumor-specific antigens [74]. However, STAT3 signaling abrogates CpG-activated immunostimulation.

Therefore, to generate a sufficient immune response, it is necessary to both stimulate TLR9 and deactivate STAT3 [76,77]. RNA interference or RNAi, is another mechanism by which STAT3 mRNA can be degraded, thereby silencing its expression. A segment of double-stranded RNA targeted to the STAT3 sequence is introduced to cells where it is metabolized to 20-21 base-pair fragments and incorporated into an RNA-induced silencing complex, RISC. The two strands of RNA are unwound, and this allows the antisense strand to bind STAT3 mRNA. RISC endonuclease activity can then cleave the target [78]. CpG-conjugated STAT3 siRNA is internalized into cells by endocytosis, where TLR9 binding facilitates the release of Dicer-uncoupled siRNA from the early endosome before acidification [79].

In vitro studies of CpG-conjugated STAT3 siRNA showed that more than 80% of mouse dendritic cells, macrophages, and B-cells were positive for uptake without transfection reagent within 60 minutes [73]. STAT3 gene silencing was achieved with maximal reduction in expression at high siRNA concentrations of 1 µM [73]. In mice bearing B16 melanoma, C4 melanoma, or CT26 colon xenograft tumors, peritumoral injection with CpG-Stat3 siRNA led to tumor regression and systemic administration reduced the number of B16 lung cancer metastases [73]. This was associated with enhanced CD8+ T-Cell recruitment and cytolytic activity in association with increased immunostimulatory cytokine and chemokine production [73,80].

Studies with murine models of AML have shown that intravenous delivery of CpG-STAT3 siRNA results in a 70-80%, CD8+ T-cell-mediated, reduction of leukemic cells in bone marrow, spleen, lymph nodes, and peripheral blood while simultaneously reducing Treg levels [81]. Combination intratumoral administration of CpG-STAT3 siRNA with radiotherapy has demonstrated complete rejection of A20 lymphoma tumors in mice and generates long-term protective immunity against the primary tumor [75]. Similarly, CpG-STAT3 siRNA inhibits tumor growth of androgen-independent prostate cancer, with a concomitant reduction of immunosuppressive MDSC levels in peritumoral lymph nodes [82].

### 1.7. Nucleic Acid-Based Agents that Act as Competitive Inhibitors of STAT3

#### 1.7.1. G-Quartet Oligodeoxynucleotides (GQ-ODNs)

**Figure 2:**
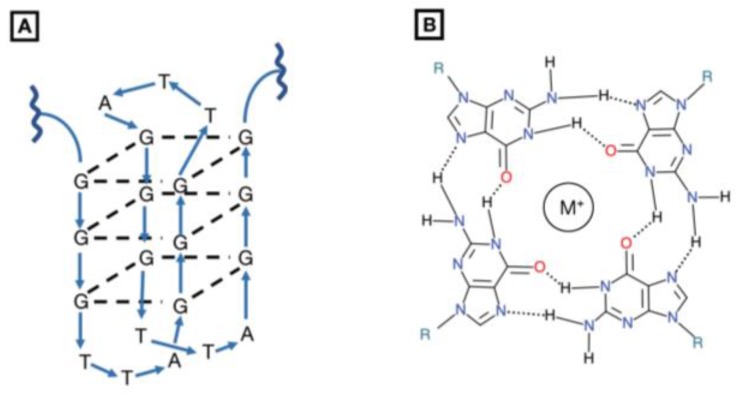
Structure of G-quartet inhibitor. (**A**) The G-quartet oligodeoxynucleotide is comprised of four guanosine macrocycles stacked on top of one another. To date, they have been used as competitive inhibitors of HIV-1 integrase but have demonstrated potential STAT3 dimerization inhibitors. With i.p. and i.v. injection in vivo mouse xenografts, G-quartets have been shown to reduce tumor growth in breast, prostate, and non-small cell lung cancers [85,86,87]. (**B**) An overhead view of a G-macrocycle demonstrates how hydrogen bonding generates a tetrad-helical structure with a monovalent cation at its core.

G-quartet oligodeoxynucleotides are macrocycles composed of four guanosine bases that, upon hydrogen-bonding, form a polyguanylate, tetrad-helical structure in the presence of a monovalent cation, usually potassium (Figure 2) [83,84]. In vivo, G-quartets are found in telomeric regions of chromosomes and in the transcriptional regulatory regions of some oncogenes [85]. Their therapeutic potential has been demonstrated as direct competitive inhibitors of HIV-1 integrase, blocking HIV-1 DNA integration into the host genome [86,87,88].

GQ-ODNs have been proposed as a class of unique, anti-cancer STAT3 inhibitors which directly destabilize the homodimerization of STAT3, and thus interfere with its DNA binding activity [89,90]. Computational analyses revealed a GQ-ODN interacts with residues Q643, Q644, N646, and N647 of the SH2 domain [91]. The folds that comprise the G-quartet intramolecular structure significantly occlude single-stranded endonuclease access to its phosphodiester linkages, resulting in an inability to cleave the oligonucleotide and in a long serum half-life [92]. However, because of their large size and charge, G-quartets cannot penetrate cell membranes and must be delivered via a polyethyleneimine (PEI) complex [93], or another delivery vehicle.

Preliminary in vitro assays demonstrated inhibition of IL-6-induced STAT3 activation in Hep2G cells incubated with the PEI-GQ-ODN T40214 (90% inhibition at 50 ng/mL). Preincubation at the same concentration inhibited the expression of anti-apoptotic mediators, with complete blockage of Bcl-XL mRNA upregulation and 50% blockage of Mcl-1 mRNA upregulation [89].

Treatment with GQ-ODN has demonstrated inhibition of STAT3 and tumor growth in in vivo models of head and neck squamous cell carcinoma (HNSCC) [94], breast cancer, prostate cancer [93], and non-small cell lung cancer (NSCLC) [95]. Inhibition of tumor growth was accompanied by a reduction in anti-apoptotic Bcl-2, Bcl-XL, Mcl-1, survivin, and VEGF in tumor tissue, and decreased expression of proliferation mediators cyclin D and c-Myc.

GQ-ODN selectivity for STAT3 is a potential consideration, given that a negative regulator of cell growth, STAT1 shares 50% sequence similarity with STAT3. Computational docking models indicate a two- to four-fold greater IC_50_ for STAT1 over STAT3 [89], and 3D analysis showed different residues involved in STAT1 and STAT3 surface interaction [96]. However, optimization of specificity for the therapeutic target is necessary for further development of clinical potential.

#### 1.7.2. STAT3 Decoys

**Figure 3:**
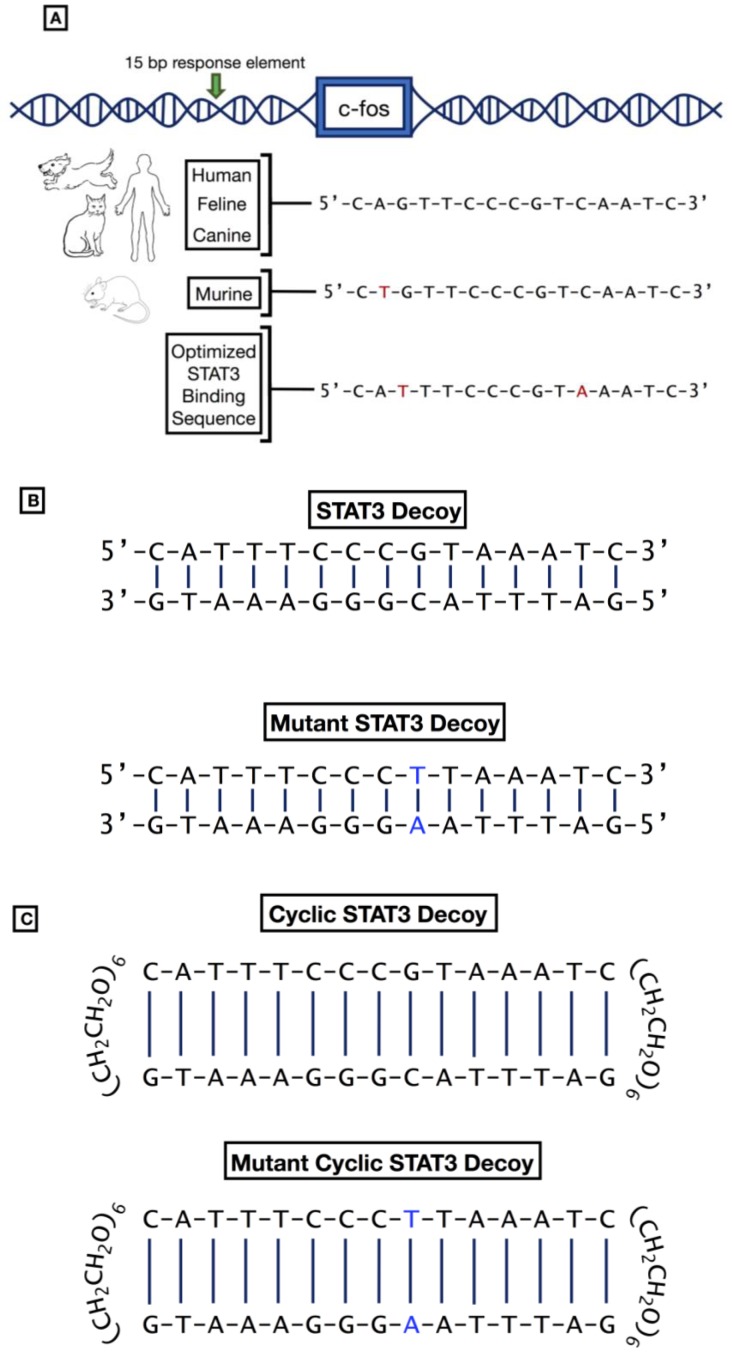
STAT3 response elements and STAT3 decoys. (**A**) The human serum-inducible element (hSIE) sequence is located upstream in the promoter region of the human *c-fos* gene and is almost perfectly conserved across human, feline, canine, and murine species. Sequence differences are shown in red. Below these is an optimized STAT3 binding sequence, with sequence modifications, shown in red, at positions 3 and 11 [52]. This allows the decoy to act as a direct competitive inhibitor of the DNA binding domain in the STAT3 molecule and thus inhibit expression of downstream anti-apoptotic and pro-proliferative signals. (**B**) Structure of the first-generation linear STAT3 decoy and mutant STAT3 decoy which is administered intratumorally in in vivo models. The location of the mutation in the mutant decoy is shown in blue. (**C**) Structure of the cyclic STAT3 decoy and mutant cyclic STAT3 decoy, with hexaethylene glycol linkages which is administered intravenously in in vivo models. The location of the mutation in the mutant decoy is shown in blue.

Once activated, STAT3 acts as a transcription factor, binding to a response element in the promoter regions of target genes to induce gene expression. Early investigation determined that STAT3 bound to a 15-base pair (bp) response element termed human serum-inducible element (hSIE) in the promoter region of the c-*fos* gene. We derived the decoy by systematically shortening the double stranded oligonucleotide to determine the smallest formulation that retained binding activity to STAT3 on gel shift assays. Figure 3A depicts the sequence of the SIE from the murine, feline, canine, and human c-*fos* genes, demonstrating nearly perfect conservation across these species. Subsequent studies identified optimal binding of STAT3 to a variant sequence of hSIE, specifically the sequence 5′-CATTTCCCGTAAATC-3′ (Figure 3A; [97]).

We synthesized the double-stranded STAT3 binding sequence (Figure 3B), the STAT3 decoy, and determined its effects when added to cells in culture [98]. As a negative control for binding specificity, a point mutant version of the decoy (termed mutant STAT3 decoy, or MT STAT3 decoy) was also synthesized and evaluated. In cell-free assays the STAT3 decoy, but not the MT STAT3 decoy, was confirmed to competitively inhibit binding of STAT3 protein to a radiolabeled decoy sequence. Fluorescently-tagged versions of both the STAT3 decoy and the MT STAT3 decoy were readily incorporated into the cytosol and nucleus of HNSCC cells and normal oral keratinocytes (NOKs) within 6 hours after treatment. Remarkably, the STAT3 decoy potently inhibited the growth of STAT3-dependent cancer cell lines, but not NOKs. The control molecule, MT STAT3, was largely ineffective against either the cancer cell lines or the NOKs.

The inhibition of cell growth resulting from treatment with STAT3 decoy was associated with induction of apoptosis and downregulation of the STAT3 target gene encoding Bcl-XL. Further investigation [99] revealed that the STAT3 decoy also inhibits the STAT1 protein, which is known to form heterodimers with STAT3. This finding was initially troubling, as STAT1 is known to have tumor suppressor activity. However, the expression or activation of STAT1 did not alter the apoptosis-inducing activity of the STAT3 decoy, indicating that the therapeutic activity of the decoy is independent of STAT1 signaling. Subsequent studies have determined that the STAT3 decoy can inhibit the growth of a broad variety of cancer cell lines, including cells representing melanoma and cancers of the bladder, brain, breast, colon, liver, lung, and ovary [98,100,101,102,103]. In addition, intratumoral injection of STAT3 decoy has been shown to inhibit the in vivo growth of breast, glioma, head and neck, lung, and ovarian xenograft tumors [98,100,101,102,103]. The anti-tumor effects of STAT3 decoy are associated with reduced tumor cell proliferation, induction of apoptosis, and reduced expression of STAT3 target genes, including the genes encoding Bcl-XL and cyclin D1 [101,104]. Based on these promising preclinical results, we conducted a Phase 0 clinical trial to assess the pharmacodynamic impact of the STAT3 decoy [105]. Intratumoral delivery of the decoy was found to downmodulate expression of Bcl-XL and cyclin D1 in the tumors of patients undergoing surgical resection of their head and neck cancer.

Fusion of the STAT3 decoy with CpG has enabled targeting of the decoy to TLR9-expressing leukemia and lymphoma cells [106,107]. Treatment of preclinical mouse models harboring leukemia or lymphoma with the CpG-STAT3 decoy resulted in potent in vivo growth inhibition of the malignant cells. Hence, the CpG-STAT3 decoy fusion molecule may provide an effective means for treating TLR9-expressing hematologic malignancies.

A major limitation of the first generation STAT3 decoy was the necessity for intratumoral injection. In preclinical studies, systemic delivery of STAT3 decoy failed to inhibit xenograft tumor growth [105], presumably due to rapid degradation of the molecule by nucleases in the blood. In an effort to produce a more stable molecule, we [105] generated a cyclic version of the decoy, using hexaethylene glycol linkages to cyclize the free ends (Figure 3C). The cyclic STAT3 decoy exhibited markedly enhanced thermal stability and a longer half-life in human serum (8 hours; [105,108]). Intravenous delivery of the cyclic STAT3 decoy has been shown to potently inhibit the growth of both non-small cell lung cancer and head and neck cancer xenograft tumors [105,109,110]. The cyclic STAT3 decoy was well tolerated in wild-type mice, with no apparent toxicity, even when delivered at a dose 20-fold higher than the maximal effective dose for growth inhibition of HNSCC xenograft tumors [110].

The therapeutic value of treatment with the STAT3 decoy, as with other STAT3 inhibitors, may be best realized in combination with other anticancer agents. In this regard, STAT3 decoy treatment has been shown to enhance the sensitivity of head and neck xenograft tumors to cisplatin [101,104]. Similarly, the STAT3 decoy heightens the response of head and neck cancer cells to bortezomib [111], ovarian cancer cells to paclitaxel [112], and leukemia cells to Adriamycin [113]. FDA IND-directed pharmacologic and toxicity studies are planned to enable a Phase I trial of the cyclic STAT3 decoy in cancer patients.

## 2. Conclusion

**Figure 4:**
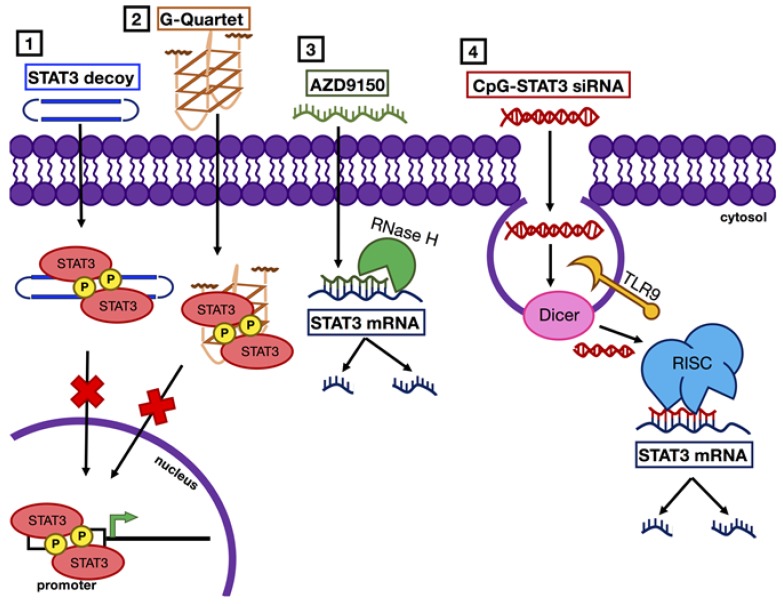
Mechanisms of STAT3 inhibition with nucleic acid-based agents. STAT3 inhibition with nucleic acid-based agents occurs via two main mechanisms: (1 and 2) STAT3 decoys or G-quartet deoxyoligonucleotides prevent STAT3 binding to promoter regions of target genes, and (3 and 4) antisense or siRNAs promote degradation of STAT3 mRNA.

Hyperactivation of STAT3 in malignant cells and tumor-associated immune cells promotes cancer survival and growth through expression of anti-apoptotic and proliferative genes, as well as cytokines that generate an immunosuppressive tumor microenvironment. Understanding the role of STAT3 in cancer has led to promising strides in the development of specific nucleotide inhibitors (Figure 4). These anti-STAT3 agents can silence STAT3 expression or directly inhibit STAT3 DNA-binding ability and have shown promising in vivo results beyond proof-of-principle studies. However, optimization of specificity, potency, stability, and delivery of these nucleotide therapeutics will be important for enhancing their therapeutic benefits in the clinic.

## 3. Discussion

Currently, there are four classes of drugs that directly inhibit STAT3: SH2 domain inhibitors; DNA-binding domain inhibitors (eg., STAT3 decoys); N-terminal domain inhibitors; and STAT3 antisense and siRNA [114]. As a whole, translation to the clinical setting has been challenged by intracellular drug delivery, selectivity for the target, and minimization of systemic toxicity. More clinical success has been achieved with inhibition of upstream Janus kinases (JAKs) using Tofacitinib, developed for the treatment of rheumatoid arthritis [115], and Ruxolitinib, developed for the treatment of myelofibrosis [116]. Still, small molecule drugs often exhibit off-target effects and a lack of targeted potency; thus, the limitations of monotherapy should be considered.

The future of STAT3 inhibition is likely to be in combination therapy with currently existing or newly developed drugs. Blocking STAT3 could result in synergistic anti-tumor effects in combination with inhibition of EGFR or other tumorigenic dysregulated transcription factors [117,118]. STAT3 inhibition resulting in resensitization to immunotherapies could pave the way for more efficacious responses to checkpoint inhibitors or other immunotherapies [119,120,121,122,123]. Looking to the future, while the role of STAT3 has been established in cancer biology, identifying tumor biomarkers that can indicate patient sensitivity to STAT3 inhibition and expansion of target inhibition to other aberrant transcription factors will be important. Research efforts should continue to search for novel applications of STAT3 inhibition and continue to pursue clinical validation of efficacy.
